# Peculiarities of the formation and subsequent removal of the circulating immune complexes from the bloodstream during the process of digestion

**DOI:** 10.12688/f1000research.14406.1

**Published:** 2018-05-21

**Authors:** Sergej B. Landa, Pavel V. Korabliov, Elena V. Semenova, Michael V. Filatov

**Affiliations:** 1 Division of Molecular and Radiation Biophysics, National Research Center , Gatchina, 188300, Russian Federation; 2 State Research Institute Center for Innovative Medicine, Vilnius, LT-01102, Lithuania; 3 Saint Petersburg State Research Institute of Phthisiopulmonology of the Ministry of Healthcare of the Russian Federation, Saint Petersburg, 191036, Russian Federation

**Keywords:** сirculating immune complexes, digestion, dynamic light scattering method, isotypes of immunoglobulins

## Abstract

**Background: **Large protein aggregates, known as circulating immune complexes (CICs), are formed in biological fluids as a result of the development of the body's immune response to various provoking factors. The kinetic characteristics of the formation and removal of immune complexes (ICs), their physical parameters, the isotypic composition of immunoglobulins (Igs) and the antigenic component of the CICs may reflect certain aspects of certain pathological and metabolic processes taking place in humans and animals. The aim of this study is to assess the kinetic characteristics of the formation and removal of the CICs that form in blood after eating. We also analyze the changes in the isotypic composition of Igs of ICs that accompany this biological process in rodents and humans.

**Methods: **We identified the CICs, which differed in size and class of Igs, using dynamic light scattering. To remove ICs from the plasma, we used immune-affinity sedimentation. Monoclonal antibodies for the Igs of different isotypes were added to the plasma samples to determine the isotypic composition of the ICs.

**Results: **A large number of ICs were formed in the blood of rats and humans after eating (food CICs). In rats, food ICs are almost immediately filtered in the liver, without circulating in the bloodstream through the body. In humans, the level of food ICs in the blood increases for 3.5 h after ingestion, then within 7–8 h their gradual removal takes place. It was found that in the process of digestion in humans, the isotypic composition of Igs in the CICs changes and becomes more diverse.

**Conclusions: **The molecular–cellular mechanisms of the formation and utilization of food CICs in humans and rodents do not match completely.

## Introduction

Circulating immune complexes (CICs) are macromolecular structures formed as a result of the specific interaction of antigens with bivalent or multivalent antibodies in biological body fluid (such as blood, saliva, urine or cerebrospinal fluid). The composition of the CICs is very diverse: CICs can contain a sufficiently large number of different components, including antigens, immunoglobulins (Igs), lipoproteins, proteins of the compliment system, proteins of coagulation, adhesion ^1, 2^.

Immune complexes (ICs) are formed due to the development of the body's humoral immune response to various provoking factors and are thought to reflect certain aspects of this process. It is believed that under normal conditions, CICs are rapidly eliminated from the bloodstream; however, with prolonged antigenic effects, their blood levels may rise ^3^. Sedimentation of CICs on the vessel walls and in basal cell membranes stimulates inflammatory processes in these tissues and cells ^4^, leading to serious tissue damage.

The content of ICs increases in the blood serum and other biological fluids during various systemic disorders, including rheumatological and autoimmune diseases, viral, bacterial and parasitic infections, malignant neoplasms and acute allergic reactions ^4– 14^. The levels of CICs change during the process of pathogenesis, as does their structure ^1, 6, 9, 15– 24^. Physico-chemical parameters (primarily size) and biological activity of IC depends on the properties of the antigen and the functional characteristics of the antibodies (including isotype, glycosylation and the ability to bind complement) ^9, 25– 28^. At the same time, information on CIC composition is more useful than information on free antigens and antibodies. Thus, a comprehensive analysis of CICs can serve as a basis for the diagnosis of various pathologies associated with defects in the functioning of the immune system, and could have prognostic value and allows for the monitoring of the effectiveness of the therapy that is used. Along with this, the detection of ICs in biological fluids, estimation of their dimensionality and determination of the isotypic composition of the Igs component in the CICs will contribute to a better understanding of the characteristics of certain metabolic processes taking place in humans and animals.

We have developed a relatively simple method of determining the CICs, differentiated by the size and class of Igs, which makes it possible to study the structural and functional features of the organization of native ICs ^29– 32^. The method is based on the analysis of the contribution of CICs, formed in biological fluids, to the light scattering recorded using a dynamic light scattering (DLS) spectrometer. This is a sensitive and accurate method, characterized by the nature of the measurement, which does not perturb the system that is being studied. Using this technique, it is possible not only to register the CICs that already exist in the blood plasma, but also to study the formation of new ICs upon the addition of exogenous materials and to estimate their distributions according to sizes, from tens to thousands of nanometres. In addition, DLS makes it possible to identify Igs of different classes and isotypes in the composition of circulating ICs without disturbing the structural integrity of the latter and at their minimum concentrations ^30, 32^.

In the present study, we used the DLS method to assess the peculiarities of the formation and subsequent removal of the CICs from the bloodstream in mammals (rats) and humans during digestion.

The aim of the present study is to assess the kinetic characteristics of the formation and removal of CICs formed in human blood after ingestion (food CICs), and analyse changes in the isotypic composition of Igs of ICs that accompany this biological process. Given that rodents, in particular rats, are often used as laboratory models in the study of digestion processes, we also performed a series of similar experiments in rats using the DLS method.

## Methods

### Determination of the scattering particles' size by means of DLS

The measurements were performed on a PLSS laser correlation spectrometer (INTOX MED LLC, Russia). We used a heterogeneous research scheme. The measurements were carried out in the band of 2,048 or 8,096 Hz. With these measurement parameters, the time taken to study one sample was about 15 min.

The particle sizes in the study samples were calculated using previously described software products included with the PLSS laser correlation spectrometer (QELS for Windows, Version 3.2, Division of Molecular and Radiation Biophysics, National Research Center “Kurchatov Institute” B.P.Konstantinov St Petersburg Nuclear Physics Institute, Russia) ^30, 33^. The measurement result is presented as a histogram of the particle size distribution (histogram of the subfraction composition), in which the abscissa is the scale of dimensions in nanometers, and the contribution to the total scattering of a sample of particles of a given size in percent is plotted along the ordinate axis. In this case, the total scattering of all particles of the sample is taken as 100%. For each sample, measurements were taken at least three times. Statistical processing of the obtained data was carried out using the regularization algorithm ^33^ with the help of QELS 3.2 software, included in the set of the PLSS laser correlation spectrometer.

See Supplementary File 1 for more information.

### Preparation of blood samples for analysis through the DLS method

In the experiment, 10 healthy men aged 24–40 years participated voluntarily. All volunteers were chosen from the staff of the Division of Molecular and Radiation Biophysics where the study was performed (National Research Center “Kurchatov Institute” B.P.Konstantinov St Petersburg Nuclear Physics Institute, Russia). All the participants conducted routine daily activities and were not treated with any medical substances for the period of the experiments. Subjects were excluded if they were taking medication during the period of the experiment. Recruitment of exclusively male volunteers was accidental, and it does not bear any significance in this study.

Use of biological material (plasma) was approved by the Ethical Committee of Polenov Russian Scienific Research Institute of Neurosurgery (Saint Petersburg, Russia) (23/H-18 of 23/10/2017). The Institute is authorized to perform research involving human participants according to guidelines from the Ministry of Healthcare of the Russian Federation (Order of the Ministry of Health of the Russian Federation “On the Ethics Committee of the Ministry of Health of the Russian Federation” of 10.07.2015 № 435n) that are in agreement with the principles expressed in the Declaration of Helsinki. Written informed consent was obtained from all donors involved to participate in the study. All blood samples were de-identified.

Blood sampling (5 ml) was done before meals and at 1.5, 3.5, 5, 7, 12 and 14 h after eating. First venous blood withdrawal was performed at 8 A.M. from fasted participants. After that, volunteers were provided with hot three-course breakfast (vegetable salad, main course (either meat or fish with garnish), either tea or coffee with a cake). The acquisition of blood plasma was carried out by the standard method ^34^. As an anticoagulant, heparin was added at a final concentration of 50 U/ml.

Removal of blood cells was carried out by centrifugation at 1,500 *g* for 15 min. The supernatant was collected and for the sedimentation of platelets and decay products of the cell elements, 1 ml was centrifuged for 30 min at 12,500 *g*. The resulting plasma sample was then diluted three times with standard isotonic phosphate buffer (pH 7.2) containing 10 mM EDTA and 1 mM sodium azide.

### Removal of ICs from blood plasma

To remove ICs from the plasma, we used immune-affinity sedimentation: an excess of protein A, immobilized on sepharose beads (Pasteur Research Institute of Epidemiology and Microbiology, Russia), was added to the blood plasma. Protein A is a protein isolated from the surface of the cell wall of *Staphylococcus aureus*, it binds the Igs of mammals well, particularly IgG ^35^.

After a 10-min incubation at room temperature, the immobilized protein A, together with the Igs bound thereto, were removed from the plasma by centrifugation of 1,000 *g* for 10 min. The supernatant, which is a blood plasma, devoid of Igs (both in free form and in the composition of ICs), was diluted three times with phosphate buffer.

### Preparation of the samples for the determination of the isotypic composition of the immunological component of IC

Monoclonal antibodies to human Igs of different isotypes were added to the plasma samples to determine the isotypic composition of the ICs formed during digestion. Adding antibodies to human Igs of different isotypes to the biological fluid results in the specific binding of ICs containing Igs of this isotype, and in the formation of even larger aggregates.

To the samples of filtered blood plasma (180 µl), prepared according to standard procedure ^30, 33^, 20 μl of a solution of monoclonal murine antibodies to human Igs of different isotypes, both in pure form and in various combinations, were added (See section “Antibody details”). In particular, we used antibodies to human IgG1, IgG2, IgG3, IgG4 and IgA. Previously, the antibodies were diluted five times with standard isotonic phosphate buffer (pH 7.2) containing 10 mM EDTA and 1 mM sodium azide.

### Antibody details

In our study, we used only monoclonal murine antibodies (MMA) to human Igs of different isotypes. All antibodies (Test kit “Diagnostic monoclonal antibodies for light and for heavy polypeptide chains of human immunoglobulins” produced according to Specifications 9398-001-11122195-08) were custom-produced for us by Polignost LLC (Saint Petersburg, Russia).

MMA to human IgA (item number: 1H9) ^36^.

Antibodies were raised in the hybridoma strain originated as the result of hybridization of myeloma PX 653A cells with splenocytes from inbred mice SJL/J immunized with the mixtures of purified myeloma proteins IgA of κ and λ types. Antibodies recognize the epitope of the α chain, and they bind effectively both with human IgA1 and IgA2. MMA were purified with affinity chromatography on protein G.

MMA to human IgG1 (item number: 2C11) ^37^.

Antibodies were raised in the hybridoma strain originated as the result of hybridization of myeloma PX 653A cells with splenocytes from inbred mice SJL/J immunized with the mixture of several human myeloma proteins IgG1. Antibodies were purified with affinity chromatography on protein G.

MMA to IgG2 (item number: 3C7).

MMA to IgG3 (item number: 5G12).

MMA to IgG4 (item number: 5C7).

The procedures of the latter three MMA productions were the same as described above with application of immunogens IgG2, IgG3 and IgG4, respectively.

### Preparation of rat blood serum samples for analysis of food CICs

In this study, six healthy adult 250–280 g male Wister strain white albino rats (10–11 weeks old) (FGUP PLZH Rappolovo, Russia) were studied. All procedures for rat care and use were conducted in accordance with the National Standard of the Russian Federation GOST R 53434-2009: Principles of good laboratory practice (introduced 01.03.2010 by Federal Agency for Technical Regulation and Metrology, published by Standardinform, Moscow, 2010). The protocol was approved by scientific committee of the Division of Molecular and Radiation Biophysics of National Research Center "Kurchatov Institute" B.P.Konstantinov St Petersburg Nuclear Physics Institute (Gatchina, Russia).

Animals were individually housed in standard polycarbonate cages (width: 206 mm, depth: 365 mm, height: 197 mm) and maintained under standard conditions of temperature (22 #177; 2°C), humidity (55 ± 10%), artificial 12-hour light–dark cycle (lights on at 8:00 A.M., lights off at 8:00 Р.M.).

The rats were divided into two groups (n=3): those that received complete dietary rations for 3 days (group one) and those that did not receive any food at all (group two). For our tests, animals were allocated to groups in a random manner. The rats in group one were fed on the nutrient extruded and granulated food designed for experimental rodents (LLC) Laboratorkorm, Russia), and they had ad libitum access to it. All animals had *ad libitum* access to water.

All efforts were made to ameliorate any suffering of animals. Rats from both groups were anesthetized for the euthanasia with diethyl ether (PJSC “Medkhimprom”, Russia) through the respiratory route by exposing animals to the ether couples for approximately 2 min in an airtight transparent acrylic jar. Dead rats were opened, and their blood was sampled from the Vena portae and from the Vena cava inferior.

Blood was collected into the standard tubes with a clot activator and incubated at 37°C for 60 min, until the clot was completely formed. The clot was separated and the serum was centrifuged at 1,500 *g* for 15 min. The supernatant was collected and again centrifuged for 30 min at 12,000 *g*. The obtained serum sample was diluted three times with isotonic PBS (pH 7.2) containing 10 mM EDTA and 1 mM sodium azide. Each analysed serum sample was divided into two aliquots, one of which served as a control. To the other, 10% (v/v) of protein A, immobilized on sepharose microspheres (20 μl microspheres were added to 180 μl serum), was added.

### Statistical analysis

Statistical processing of the obtained data was carried out using the regularization algorithm ^33^ through application of software QELS 3.2, included in the set of the PLSS laser correlation spectrometer (QELS for Windows, Version 3.2, National Research Center "Kurchatov Institute" B.P.Konstantinov St Petersburg Nuclear Physics Institute, Russia).

In each individual experiment, the error in determining the mean hydrodynamic size of the light-scattering particles in the study samples of biological fluids can be several tenths of a percent (and this value is reproduced when the experiment is repeated on the same sample). The error in determining the mean hydrodynamic size of the light-scattering particles on a series of equally prepared samples is usually higher by an order of magnitude.

To analyze the dynamic of accumulation of food ICs and their subsequent dissipation in blood serum samples from 10 volunteers, applying DLS, we calculated mean and standard deviation (SD) at several time points for the following parameters of interest: hydrodynamic radius of the scattering particles, their contribution to the light scattering. Mean inter-group difference was calculated with 99% confidence intervals.

The same calculations were carried out to evaluate isotypic composition of immunoglobulins of human CIC after eating.

Computations were performed using MS Excel 2007 standard statistical functions (Statistical Package Microsoft Office 97 for Windows, Redmond, USA).

## Results

### Peculiarities of the formation and utilization of the CIC during digestion in rats

In the experiment, two groups of rats participated: the first group of animals received a complete food ration for 3 days and the second was starved. For the analysis by the DLS method, serum from the rat portal vein of the liver and from the inferior vena cava was used.

To reveal the CICs that formed during the process of digestion, we applied the approach associated with the selective removal of Igs and their complexes from the analysed blood sample using protein A immobilized on sepharose beads, followed by centrifugation ^31, 32^. Comparison of the histograms of the subfractional composition of particles of blood serum samples, those processed with protein A and those not processed obtained by the DLS method, allows for the recording of the presence or absence of a fraction of large scattering particles, the hydrodynamic radius (R _h_) of which corresponds to the size of ICs.

**Figure 1.**
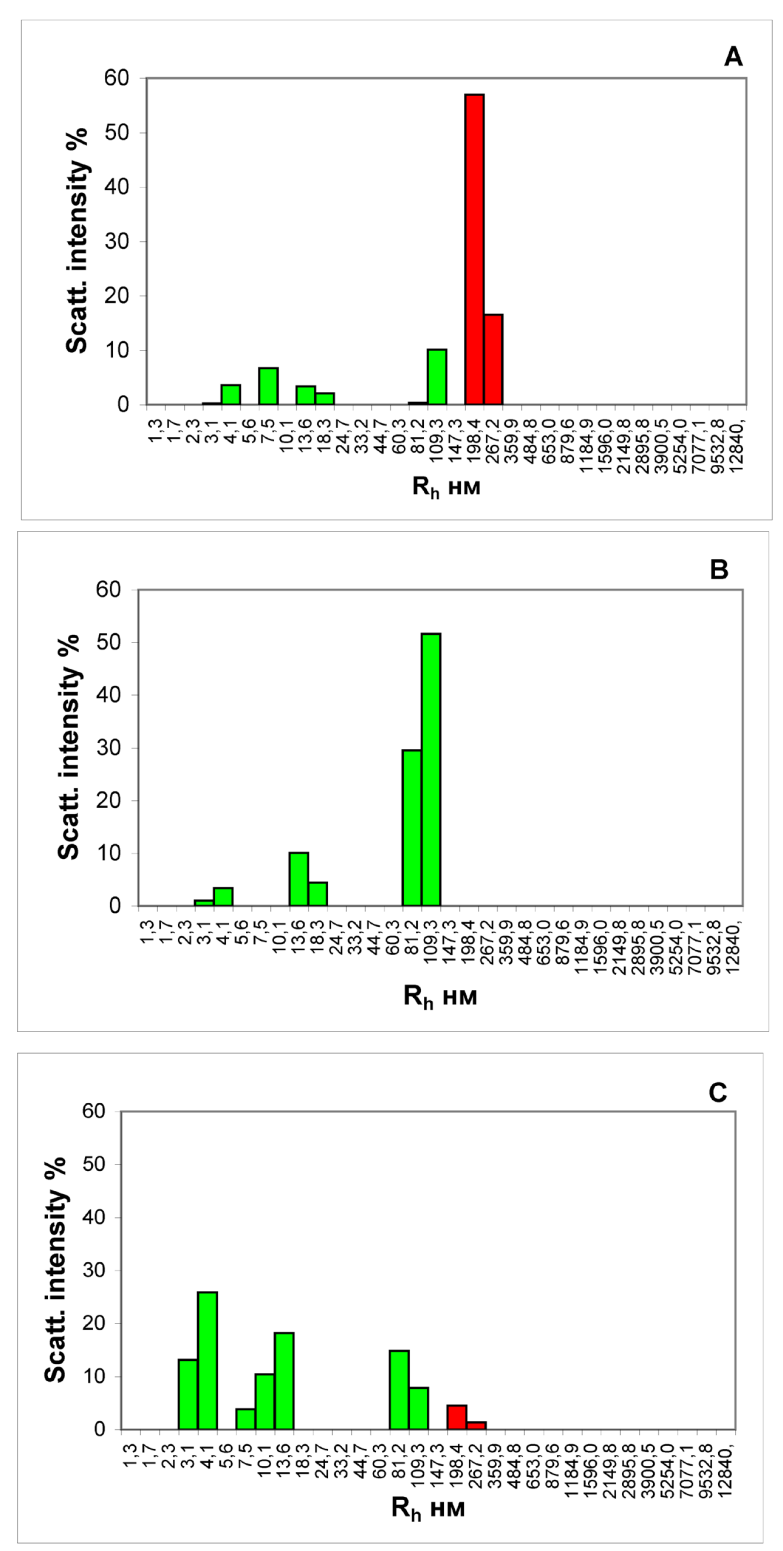
Histograms of the particle distribution by the size of the blood serum samples from the portal vein of liver of the rat that received a wholesome food ration for 3 days ( **A** and **B**) and the rat that starved for 3 days ( **С**). The figure presents the results of analyses of the blood serum samples each taken from one most representative rat from each experimental group. ( **A**, **С**) Samples of native serums. ( **B**). Sample of serum, treated with protein A, immobilized on the agarose microspheres. The *x*-axis is the hydrodynamic radius of the particles in nm, the *y*-axis is the contribution to the scattering in %.

The results from analysis of the blood serum from the portal vein of rats that received a full-fledged food ration are shown in Figure 1. It can be seen that in the blood of the animal, there are large protein aggregates, with an R _h_ of the order of 200–300 nm ( Figure 1A), disappearing after treatment of the serum with protein A immobilized on sepharose microspheres ( Figure 1B). We have identified these protein aggregates as food CICs.

The portal vein in animals is a venous trunk, through which blood from the stomach and intestine passes into the liver. Thus, in the blood of a satiated rat that is coming from the digestive tract to the liver, a substantial amount of CICs are observed ( Figure 1A). In the blood serum taken from the portal vein of the rats that were starved for three days, the CICs are practically absent ( Figure 1С).

**Figure 2.**
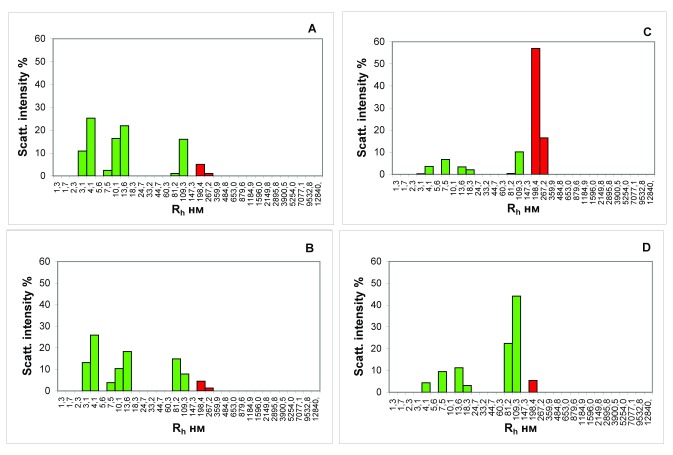
Histograms of the particle size distribution of the serum samples of hungry rats ( **A** and **B**) and rats receiving a wholesome food ration ( **С** and **D**). Blood was taken from the portal vein of the liver ( **A** and **С**) and the inferior vena cava ( **B** and **D**). *x*-axis, the hydrodynamic radius of the particles in nm; *y*-axis, the contribution to the scattering in %.

The results of research of the blood samples from the portal vein of the liver and from the inferior vena cava of the fed and hungry rats also differ substantially; this is evidenced by the data presented in Figure 2.

The inferior vena cava collects venous blood from most of the abdominal and pelvic organs, the walls of these cavities and lower limbs. By comparing blood from the portal vein of the liver and inferior vena cava, it is possible to obtain the information about changes in blood composition due to the filtration processes performed by the liver.

Comparison of Figure 2A and B shows no changes in the blood serum particle subfractional composition of the rats that starved for 3 days, before and after filtration in the liver. The contribution to the scattering of the fraction of particles matching the ICs in the blood that enters the liver of their gastrointestinal tract (6.1%, Figure 2A) practically matches the analogous indicator in the blood after the passage through the liver (5.8%, Figure 2B) in the starving rats.

Contrary to that, the contribution to the scattering of particles with the R _h_ that matches the ICs in the blood sample, taken from the portal vein of the rats that received a normal food ration is higher by more than an order of a magnitude than the contribution to scattering of particles of the same size in a blood sample taken from the inferior vena cava (73.3%, Figure 2C versus 5.4%, Figure 2D).

Thus, the presented results allow us to state that in the process of digestion, a substantial number of ICs that diffuse into the blood are formed in the intestine of rats. These food ICs are completely utilized in the liver.

### Peculiarities of the formation and utilization of the CICs during digestion in humans

In total, 10 healthy men participated in the experiment. Blood sampling was done before meals and at 1.5, 3.5, 5, 7, 12 and 14 h after eating.

We determined the total contribution to the scattering of the fraction of ICs and the isotypic composition of the Ig component of the food CICs. Analysis of the results showed that the level of ICs in all inspected donors gradually increase in the blood, reaching its maximum at 3.5 h after eating.

**Figure 3.**
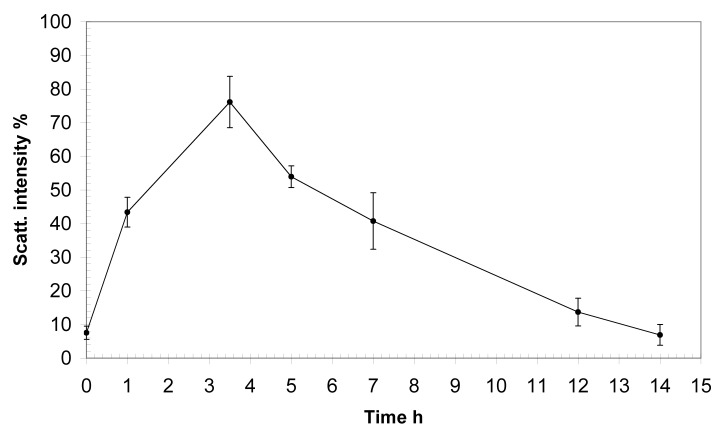
Dynamics of the accumulation of immune complexes in the blood of healthy young men after eating. *x*-axis, time after eating; *y*-axis, contribution to the scattering of the fraction of food ICs in %.

As seen in Figure 3, the base level of the contribution to the scattering of the fraction of particles matching the ICs in the serum was less than 10%. The maximum IC contribution to the scattering was observed after 3.5 h, when it reached almost 80%. Then, the IC fraction contribution of the scattering decreased, reaching the basal level 14 h after eating.

Table 1 presents the subfractional composition of the particles (the fraction that matches the ICs) of the serum of all the examined donors before eating and at the point of the maximum accumulation of ICs after eating (after 3.5 h). Before meals, there is an insubstantial variation in the size of the recorded ICs and a more substantial difference in the contribution to the scattering of IC fractions in the donors. At 3.5 h after eating, when the level of food complexes in the blood reaches its maximum, the sizes of the complexes for different donors practically match.

**Table 1.** Size of immune complexes and their contribution to the scattering in the examined donors before and 3.5 h after eating.

Donor	Before eating		3.5 h after eating	
	Size, nm	Contribution to scattering, %	Size, nm	Contribution to scattering, %
D10	109	8.5	122	78.6
A10	119	7.1	120	78.6
A05	109	2.6	121	78.1
B03	116	8.3	120	78.8
C04	127	4.1	123	79.8
B01	121	3.7	121	78.5
A18	123	6.9	122	79.3
FF	115	7.3	120	78.7
A12	118	8.5	122	78.3
C14	109	2.6	121	78.6
Mean	116.6	5.9	121.1	78.7
SD	6.0	2.4	0.95	0.5

### Analysis of changes in the isotypic composition of the Ig component of the CICs during the process of digestion in humans

As reported previously, the addition of antibodies to Igs of different isotypes to the blood plasma results in the specific binding of ICs, containing Igs of this isotype, and the formation of even larger protein structures. The appearance of a new fraction of light-scattering particles with a large R _h_ on the histograms of the subfractional composition of particles, obtained by the DLS method, attests to the presence of this variation of Igs in the analysed ICs. According to the contribution to the scattering of these high-molecular-weight protein aggregates to the total contribution of all ICs, it is possible to determine the proportion of ICs containing Igs of this isotype ^30, 32^.

**Figure 4.**
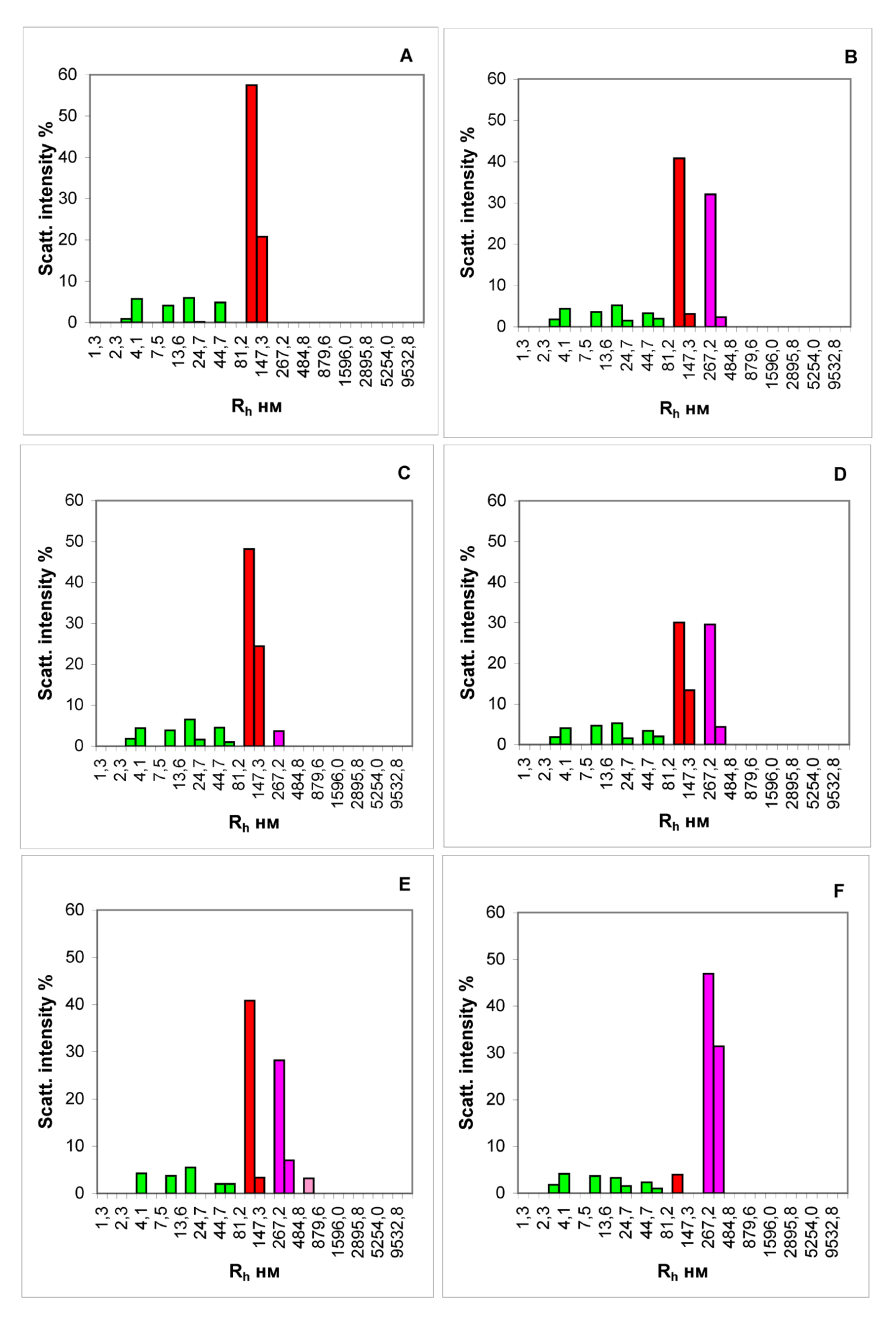
Isotypic composition of immunoglobulins of blood immune complexes (ICs) of a healthy donor 3.5 h after ingestion. ( **A**) The total contribution of ICs. ( **B**) The contribution of isotype IgG1 complexes. ( **C**) The contribution of isotype IgG3 complexes. ( **D**) The contribution of IgA complexes. ( **E**) The contribution of complexes of IgG1 and IgG3. ( **F**) The contribution of complexes of IgG1 and IgA. *x*-axis, the hydrodynamic radius of the particles in nm; *y*-axis, the contribution to the scattering in %.

Analysis of the isotypic composition of the Ig component of the CICs showed that before eating, IgG1 Igs are predominantly present in CICs of all of the examined donors. After 3.5 hours after ingestion, the isotypic composition of Igs in the IC expands ( Figure 4) along with the complexes of IgG1, the number increases to 33.1 ± 1.6% (mean ± SD). ( Figure 4B), an insignificant number of IgG3 complexes (4.1 ± 0.9%) appears ( Figure 4C), and the content of IgA complexes becomes predominant (42.0 ± 0.8%) ( Figure 4D).

The results of the isotypic composition of Igs of the blood ICs of all examined donors are presented in Table 2.

Thus, the process of digestion in humans is accompanied by the formation of the CICs, the level of which reaches a maximum value the blood after 3.5 h. Initially, ICs mainly contains the IgG1, then a set of Ig isotypes in the composition of food ICs becomes more diverse. After 3.5 h, a gradual decrease in the amount of food ICs in the blood begins, and 14 h after eating, their concentration drops to the values corresponding to the initial level (before eating).

**Table 2.** Isotypic composition of immunoglobulins from blood circulating immune complexes of the examined donors 3.5 h after eating.

Donor	IgG1		IgG3		IgA		IgG1+IgG3		IgG1+IgA	
	Size, nm	Contribution, %	Size, nm	Contribution, %	Size, nm	Contribution, %	Size, nm	Contribution, %	Size, nm	Contribution, %
D10	267.2	33.1	267.2	3.7	267.2	40.7	317.4	36.8	653.0	3.7
A10	267.2	32.4	267.2	3.9	267.2	41.4	311.0	36.3	653.0	3.1
A05	267.2	34.3	267.2	2.7	267.2	41.0	312.1	37.0	653.0	3.3
B03	262.0	33.9	247.3	5.1	267.2	42.1	304.0	39.1	653.0	2.7
C04	267.2	28.7	267.2	4.1	267.2	43.0	327.1	32.9	653.0	2.9
B01	287.1	33.2	252.5	4.6	267.2	42.3	323.3	37.9	653.0	2.7
A18	267.2	33.3	267.2	4.7	267.2	42.4	316.8	38.0	653.0	4.0
FF	267.2	34.0	267.2	3.3	267.2	42.1	325.8	37.3	653.0	3.0
A12	286.2	34.4	254.7	5.6	267.2	42.2	321.5	40.0	653.0	3.2
C14	282.4	33.3	247.2	3.6	267.2	42.4	303.0	36.8	653.0	3.5
Mean	271.6	33.1	260.5	4.1	267.2	42.0	316.2	37.2	653.0	3.2
SD	10.0	1.6	8.9	0.9	0.0	0.7	8.5	1.9	0.0	0.4

The raw light scattering data for the subjects at each time point, and for the starved and fed rats Click here for additional data file. Copyright: © 2018 Landa SB et al. 2018 Data associated with the article are available under the terms of the Creative Commons Zero "No rights reserved" data waiver (CC0 1.0 Public domain dedication).

## Discussion

We recorded a notable fact: numerous macromolecular structures that efficiently scatter light are formed in the plasma after eating. On the basis of the data presented herein, the bulk of high-molecular-weight aggregates of blood plasma, arising *de novo* during digestion, is formed by Igs of different isotypes and represents ICs ( Figure 4). It is obvious that the appearance of ICs in the blood after ingestion should be accompanied by their subsequent removal through cellular macrophage activity, otherwise their permanent accumulation would be observed. We analysed the kinetic characteristics of complexation during digestion in 10 healthy men. All the examined donors had similar temporal characteristics of the appearance, accumulation and removal of food ICs from the bloodstream. The number of newly formed protein aggregates in the peripheral blood increased by more than 5 times fairly quickly, within about 1 h of eating. Later, within several hours, the level of the CICs gradually increased, reaching its maximum after 3.5 h, after which a smooth decrease in the concentration of food ICs begins. After 7-8 h from that moment, characterized by the highest concentration of CICs in the blood, their level returns to the initial state ( Figure 3). Thus, the cycle of appearance and removal of food ICs in humans lasts approximately 12 h.

We also performed a series of experiments in rats using the DLS method. We compared the composition of the blood (specifically the presence of the CICs) of rats that were kept hungry for 3 days with similar indicators of fed animals. For these purposes, samples of blood from the portal vein and inferior vena cava of the liver of rats were analysed.

By analysing the blood from the portal vein, we get information about the composition of the blood before it passes through the liver filtration barriers. The results of our studies show that the digestive process in the rat is accompanied by the appearance of a large protein aggregates with an R _h_ of the order of 200–300 nm, in the blood taken from the portal vein of a satiated animal, which we identified as ICs ( Figure 1). That is, in a satiated rat in the blood coming from the digestive tract to the liver, a substantial amount of the CICs are observed ( Figure 1A). In the serum of the rats that were not fed for 3 days, taken from the portal vein, the CICs are practically absent ( Figure 1C).

The lower vena cava collects the venous blood from most of the abdominal and pelvic organs, including the blood that has been filtered out by the liver. By comparing the blood from the portal vein of the liver and the inferior vena cava, we can comprehend the changes in the composition of the blood, due to the detoxification carried out by the liver. Virtually no changes in the composition of the blood samples of hungry rats before and after passage through the liver purification filters, were recorded. In this case, the contribution to the scattering of particles with an R _h_, corresponding to the ICs, in a blood sample, taken from the portal vein of rats receiving a normal food ration, greatly exceeds the contribution to the scattering of particles of the same size in a blood sample taken from the inferior vena cava ( Figure 2C and D). Thus, we can conclude that the ICs formed during the digestion in rats almost immediately enter the liver with the bloodstream, where they are completely utilized.

The obtained data show that the mechanisms of formation and removal of food ICs in humans and rodents are different. If in rats, food ICs are almost immediately filtered in the liver without circulating in the future with the blood flow through the body, then CIC level in the human blood rises within 3.5 h after eating, after which the blood cleaning of food ICs takes place for a fairly long time (7–8 h).

The formation of numerous ICs as a result of food intake in the blood plasma is quite unusual. Ideally, during the digestion, the products of complete effective splitting of organic substances should easily leave the body in the form of water-soluble low-molecular-weight fractions, without clogging or overloading the filtering and excretory systems. However, this is not the case in practice: macromolecular structures consisting of food fragments are found in peripheral blood. The free transition of large organic molecules and/or their blocks in unsplit form through the walls of the digestive tract (primarily the intestine) into the bloodstream is called transcytosis ^38– 40^.

The causes and biological relevance of transcytosis during digestion are not clear. However, it is obvious that it induces a local immune response. The data on the growth of CICs in the human blood during the process of digestion presented herein indirectly confirm this. The digestive tract has a number of barrier functions that protect the body from pathogens that enter the human body from the external environment. Nonspecific barrier mechanisms include the antibacterial properties of saliva, bile and pancreatic juice, the proteolytic activity of secrets, intestinal motorics and the characteristic structure of the surface of the intestinal mucosa. This first line of defence against foreign microorganisms and toxic substances is supplemented by a specific immune defence system localized in the digestive tract, which constitutes an important part of the overall multi-component human immune system. The local immune system of the digestive tract provides two main functions: recognition and induction of tolerance to food antigens and a blocking effect on pathogens ^41^. The joint coordinated actions of epithelial cells, immunoreactive mucosal cells, antibodies and synanthropic microbiota form an adaptive immune response ^42^.

One of the important functions of the mucous membranes is the production of so-called secretory IgA (sIgA), the level of which is much higher in various parts of the gastrointestinal tract than those of other classes of Igs ^43^. sIgA participates in the protection of mucous surfaces from toxins, viruses and bacteria. The polymeric nature of sIgA provides significant advantages in the fight against foreign antigens: sIgA promotes the destruction of pathogens by proteolytic enzymes while remaining intact ^44^.

sIgA molecules are involved in protection against harmful micro-organisms and exogenous agents through a mechanism called immune exclusion ^45^. In addition, as a result of the interaction of sIgA with the cells of Peyer’s patches, the intestinal transepithelial transfer of Igs from the intestinal lumen to the lymphoid tissues takes place in the intestines ^46^. Finally, in the IC, with antigens in the mucous membranes, sIgA causes a systemic reaction accompanied by inducing the production of anti-inflammatory cytokines and limiting the activation of dendritic cells ^47^. In terms of humoral immunity at mucosal surfaces, sIgA appears thus to combine properties of a neutralizing agent (immune exclusion) and of a mucosal immunostimulant inducing effector immune responses in a noninflammatory context favorable to preserve local homeostasis of the gastrointestinal tract ^48, 49^.

Evaluation of the isotypic composition of the food ICs, in our opinion, will contribute to a better understanding of the molecular-cellular mechanisms that support the local immune system of the digestive tract. Analysis of the immunoglobulin component of the ICs showed that before eating, IgG1 was the predominant Ig present in the CICs of all examined donors. During the process of digestion, the isotypic composition of immunoglobulins in the CICs expanded ( Figure 4): the number of IgG1 complexes is substantially increased ( Figure 4B) and a small number of complexes of IgG3 appear ( Figure 4C). In this case, the content of IgA complexes becomes predominant ( Figure 4D).

The phenomenon we observed with the help of the DLS method is likely to be a reflection of the features of the metabolic process in a person. This is accompanied by the entry into the blood of relatively large molecular structures, which are not completely digested in the oral cavity and the gastrointestinal path by digestive enzymes into monomers. By binding with the antibodies, they form ICs of considerable size. Basing on the presented results we arrive at the assumption that digestion in humans is accompanied by transcytosis from the intestine to the blood of not only individual molecules of Igs, vitamins, enzymes or large protein aggregates of incompletely digested food ^50^, but also ICs formed in the digestive tract as a result of activation of the local immune system (prevailing share of ICs with IgA in their structure) ( Figure 4). Apparently, subsequent elimination of these food ICs from blood stream is performed by mononuclear phagocytes ^51^. Presumably, in this case we are observing an alternative or additional mechanism of digestion that is activated under certain circumstances that are yet unknown.

## Conclusions

The mechanisms of the formation and removal of food CICs in humans and rodents are different. In humans, the concentration of food ICs in the blood starts rising fast almost instantly after food intake. It reaches the maximum in approximately 3.5 h, being 7 times its initial level. The ICs concentration returns to the initial level after a further 7–8 h through a gradual decrease. In the process of digestion in humans, the isotypic composition of Igs in the food CICs changes and becomes more diverse with time after eating. The food ICs formed during the digestion in rats almost immediately enter the liver with the bloodstream, where they are completely utilized.

## Data availability

The data referenced by this article are under copyright with the following copyright statement: Copyright: © 2018 Landa SB et al.

Data associated with the article are available under the terms of the Creative Commons Zero "No rights reserved" data waiver (CC0 1.0 Public domain dedication). 

**Dataset 1. The raw light scattering data for the subjects at each time point, and for the starved and fed rats.** DOI: 10.5256/f1000research.14406.d203311 ^52^.

## Supplementary material

**Supplementary File 1. Determination of particles size by means of the method of dynamic light scattering.**
