# Supplementary material for: Peculiarities of the formation and subsequent removal of the circulating immune complexes from the bloodstream during the process of digestion
Source: F1000Res. 2018 May 21;7:618. [Version 1] doi: 10.12688/f1000research.14406.1 (PMC6058468; doi:10.12688/f1000research.14406.1)
Supplement: Supplementary file 2 [file f1000research-7-15679-s0001.tgz › cdab7415-fbc8-44d6-a60c-e2a5f97710e8.docx]

**Supplementary File 1: Determination of particles size by means of the method of dynamic light scattering**

Statistical processing of the obtained data was carried out with the help of software, included in the set of the laser correlation spectrometer "PLSS" ("INTOX MED" LLC, Russia; Registration certificate for medical products of the Ministry of Healthcare of the Russian Federation No. РZN 2014/1650 of June 9, 2014). The device was developed and created in the Division of Molecular and Radiation Biophysics, National Research Center "Kurchatov Institute" B.P.Konstantinov St Petersburg Nuclear Physics Institute, and approved by the Committee for New Medical Technology of the Ministry of Healthcare of the Russian Federation for determining the size of microparticles in biological fluids (Certificate RU. C. 39.003. A No. 5381).

**Method**

The method of dynamic light scattering (DLS) is based on the interaction of monochromatic coherent radiation with light-scattering particles of the liquid that is being studied. Information on all the dynamic processes in the system that is studied is contained in the spectrum of fluctuations of light, scattered by particles in the solution.

In the case of scattering on a particle, performing a chaotic Brownian motion, the spectrum of the scattered light has the form of a dome-shaped Lorentz curve. The spectrum of photocurrent fluctuations at the output of the photodetector (or the correlation function) matches the spectrum of scattered light and is also described by Lorentzian.

The parameter, characterizing the width of the spectral contour (half-width at half-height of the Lorentzian), is called the diffusion broadening Г or the half-width of the spectrum. This parameter is related by a simple mathematical relation with the diffusion coefficient of the scattering particle D. By measuring the frequency at which the amplitude of the spectrum decreases two times when compared to the amplitude at zero frequency, or the time for which the correlation function decreases *e* times, we obtain the value of Г, and then, knowing the scattering angle *Ѳ*, the wavelength *λ* of the light that falls onto the system that is investigated and the refractive index *n* of the medium, we find the coefficient of diffusion diffusion *D* by formula

D = *Г/q^2^*, where
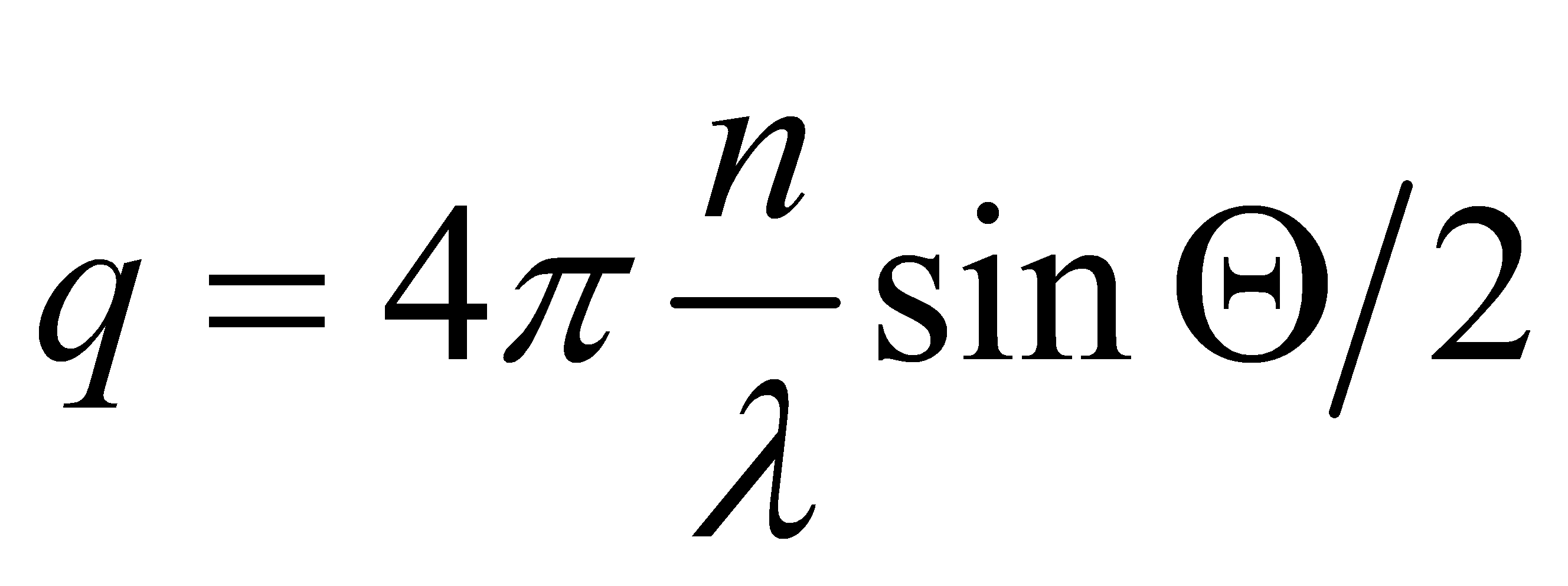
 (1)

By combining the previous expression (1) with the Stokes-Einstein formula:


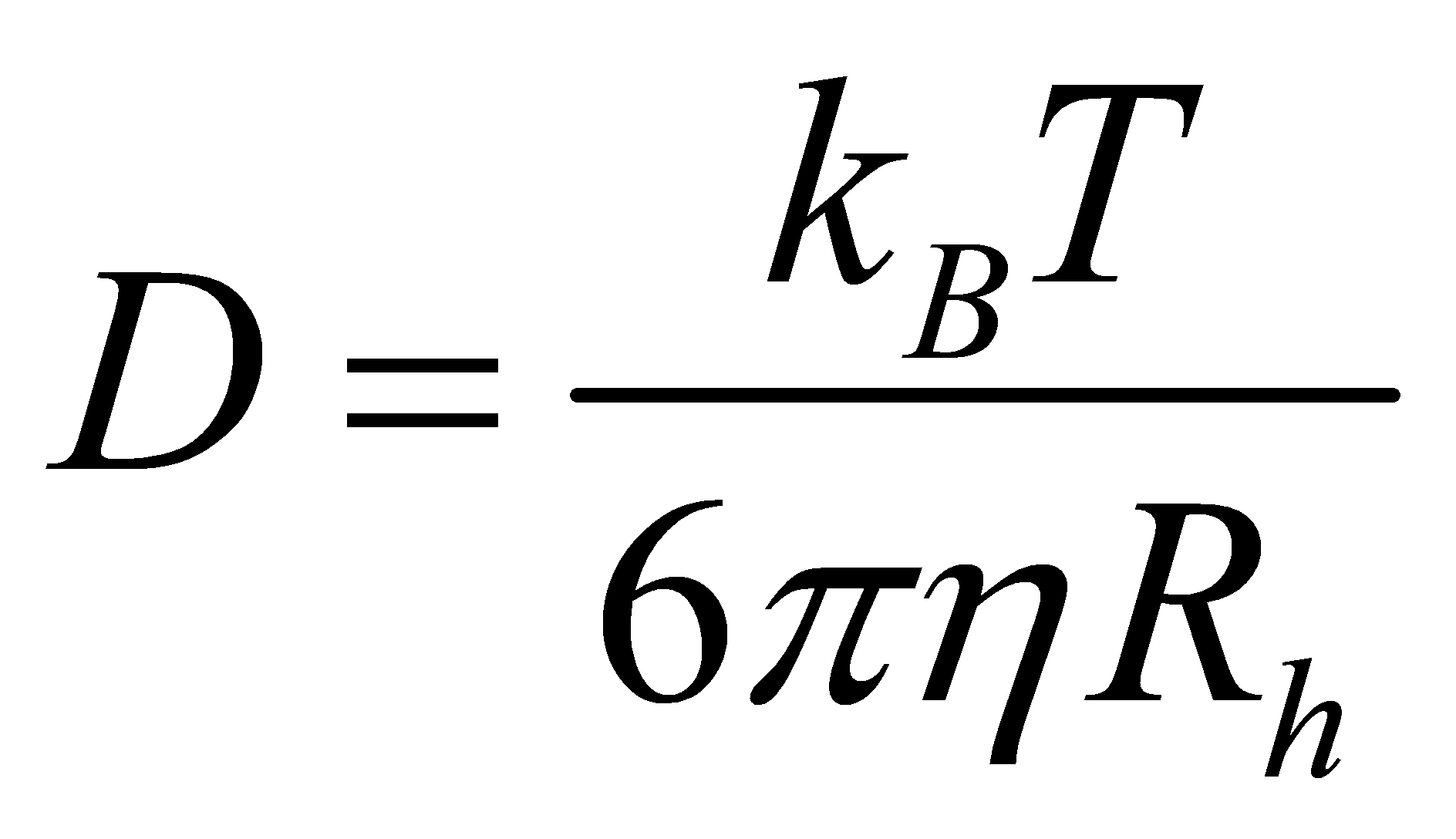
 (2)

where *η* – solution viscosity; *k_B_* – Boltzmann's constant; *T* – absolute temperature;
*R_h_* – hydrodynamic radius of a particle, we will get


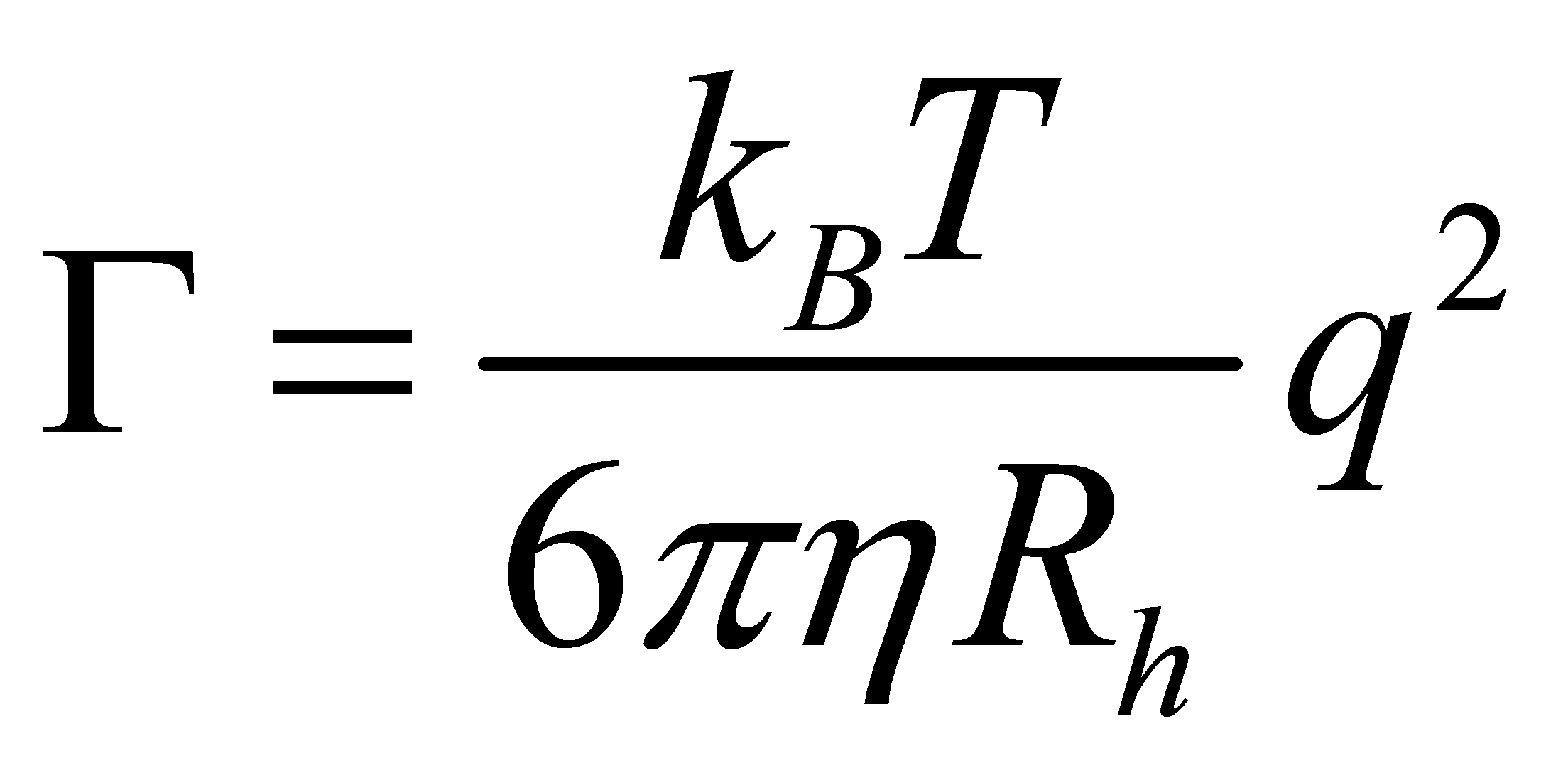
 (3)

For example, for particles in the water at a temperature of 20 °C, for λ = 632.8 nm (helium-neon laser) formula (3) takes the form


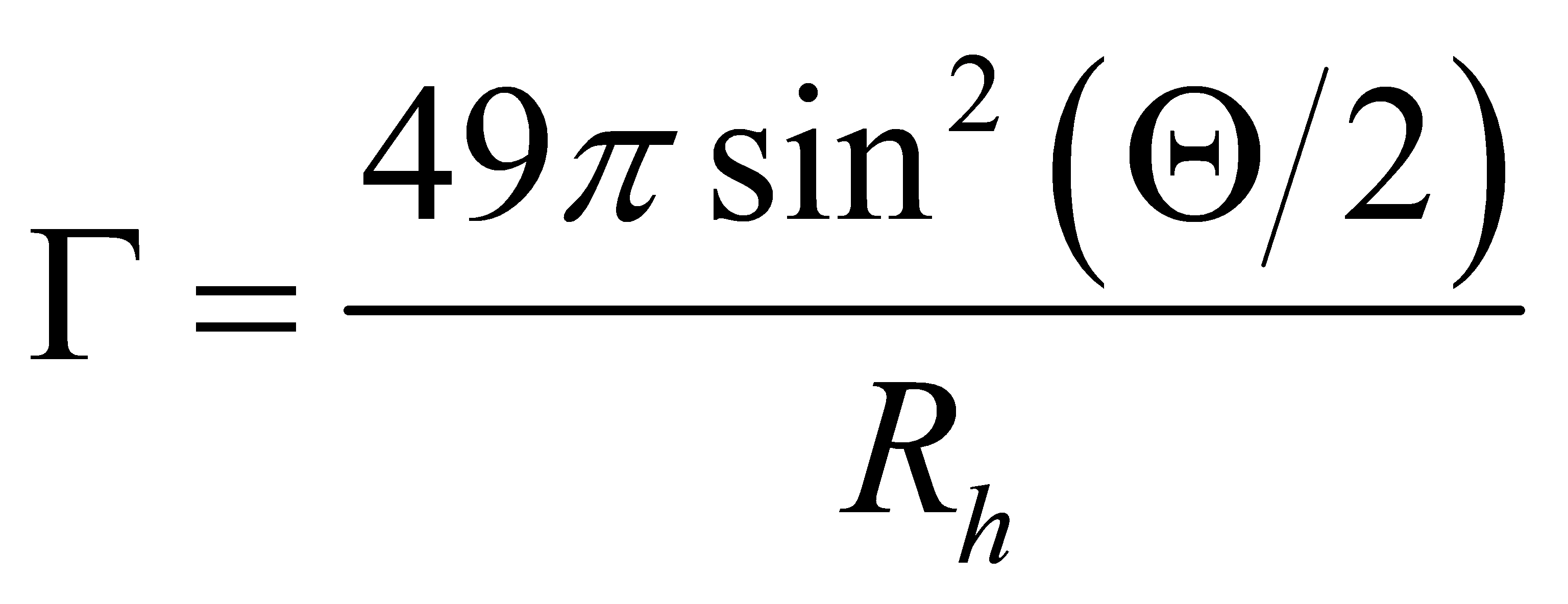
 (4)

where *R_h_* is expressed in micrometers.

Thus, by knowing the diffusion coefficient of the object that is studied, we easily determine its size [Lebedev AD, Levchuk JuH, Lomakin AV, Noskin VΑ. Laser correlation spectroscopy in biology. Kiev: Naukova Dumka, 1987].

It should be emphasized that, unlike classical spectroscopy, where light first passes through a spectral instrument and then is detected by a photodetector, in the case of DLS use, the light is first recorded by a photodetector, and then the photocurrent is analyzed by a spectral instrument.

The spectrum of fluctuations of the photocurrent of scattering particles of different sizes, contained in polydisperse biological fluids, is the sum of Lorentzians, the half-width of which characterizes the hydrodynamic radius of the particles, and the amplitude is the contribution of particles of a given size to the total scattering of the sample. It is obvious that analysis of the photocurrent fluctuation spectrum is a complicated mathematical procedure. The most well-known method for solving this type of problem is the Tikhonov's regularization method. This algorithm (with some changes) is implemented in the software that we use [Braginskaja TG, Kljubin VV. Solution of the inverse problem of optical mixing spectroscopy by Tikhonov's regularization method. Preprint LNPI AS USSR. Leningrad, 1983, p. 42].

The solution of the inverse problem by the regularization method makes it possible to reconstruct a histogram of the particle size distribution, contributing to the light scattering, from the photocurrent fluctuation spectrum. Such histogram consists of columns, the height of which reflects the percentage of contribution to the light scattering of the components of the analysed biological fluid. The modern measuring technology makes it possible to accurately measure the spectrum of photocurrent intensity fluctuations in the frequency band from few Hertzes to 20-50 kHz, this in turn makes it possible to estimate the particle sizes in the range from nanometers (2-5 nm) to tens of micrometers.

We note that the presence of particle aggregates and impurities in the sample that is analyzed, the inaccuracy of the model representations of the hydrodynamic form, and the contribution of internal dynamics - all of this does not allow to determine the size, much less the size distribution function, with an accuracy that is close to the statistical accuracy of the laser correlation spectroscopy itself. Therefore, although in each individual experiment the error in determining the average hydrodynamic size of the scatterers can be several tenths of a percent (and this value is reproduced when the experiment is repeated on the same sample), the scatter of the analysis results on a series of even equally prepared samples is usually an higher by an order of magnitude.

In the presented work, the measurements were performed on a laser correlation spectrometer - "PLSS" manufactured by "INTOX MED" LLC. We used a heterogeneous research scheme, in which the fronts of two beams (scattered and reference) are combined on the photodetector. As the reference beam, the laser radiation that irradiates the system that is studied, was used. Taking into account that it is easiest to achieve a high efficiency of heterodyning for light scattered at 90° [Lebedev AD, Ivanova MA, Lomakin AV, Noskin VA. Heterodyne quasielastic light-scattering instrument for biomedical diagnostics. Appl Opt. 1997; 36(30): 7518–22], in our experiments this particular measurement option was used.

When choosing the measurement modes (the spectrum band and the number of accumulation cycles), we followed the considerations on the most reliable recovery of the particle size in the range of interest. The measurements were carried out in the band of 2048 Hz or 8096 Hz. According to [Braginskaja TG, Kljubin VV. Solution of the inverse problem of optical mixing spectroscopy by Tikhonov's regularization method. Preprint LNPI AS USSR. Leningrad, 1983, p. 42], the particle sizes are most accurately reconstructed, when the measurement band is not less than 10 Lorentzian half-widths. For particles with the sizes that range from 0.1 to 1.5 μm, the Lorentzian half-width lies in the range from 20 to 500 Hz, the optimal band will be 8096 Hz at a resolution of 4 Hz per channel (2048 pixels of the spectrum). The above conditions require that the "nonsmoothness" of the spectrum was not higher than 0.03. Since the fluctuations of the experimental points in the spectrum are almost always of random nature, we can assume that the value of "nonsmoothness" is inversely proportional to the square root of the number of copies of the total spectrum. Thus, the number of copies of the accumulation should be at least 1000. To exclude unknown factors, we chose this value to be 2000. With such parameters of measuring, the time taken to study one sample was about 15 minutes.

The particle sizes in the study samples were calculated according to the software products that were described earlier [Landa SB, Filatov MV, Arutiunian AV, Varfolomeeva EV Study of plasma megamolecular complexation by laser correlation spectroscopy Klin. Lab. Diagn. 2008;4:37-41]. The measurement result is presented as a histogram of the particle size distribution (histogram of the subfraction composition), in which the abscissa is the scale of dimensions in nanometers, and the contribution to the total scattering of a sample of particles of a given size in percent is plotted along the ordinate axis. In this case, the total scattering of all particles of the sample is taken as 100%. For each sample, measurements were made at least 3 times; the data that was obtained as a result of regularization, was averaged.
