# Supplementary material for: Peculiarities of the formation and subsequent removal of the circulating immune complexes from the bloodstream during the process of digestion
Source: F1000Res. 2018 May 21;7:618. [Version 1] doi: 10.12688/f1000research.14406.1 (PMC6058468; doi:10.12688/f1000research.14406.1)
Supplement: Peer review history

# Peer review: Referee response for version 1

**DOI:** 10.5256/f1000research.15679.r35397

**Published:** 2018-07-20

**Authors:** Vadim Govorun

**Affiliations:** 1 Federal Research and Clinical Center of Physical-Chemical Medicine of Federal Medical Biological Agency (FRCC РСМ), Moscow, Russian Federation

This manuscript 'Peculiarities of the formation and subsequent removal of the circulating immune complexes from the bloodstream during the process of digestion' by Sergej B. Landa *et al* describes the molecular–cellular mechanisms of the formation and utilization of food CICs in humans and rodents. Circulating immune complexes (CICs) are macromolecular structures formed as a result of the specific interaction of antigens with bivalent or multivalent antibodies in biological body fluid. Authors have developed a relatively simple method of determining the CICs, differentiated by the size and class of Igs, which makes it possible to study the structural and functional features of the organization of native ICs. They found the fact numerous macromolecular structures that efficiently scatter light are formed in the plasma after eating and the mechanisms of formation and removal of food ICs in humans and rodents are different. This manuscript provides a new information about special metabolic process in a person, which accompanied by the entry into the blood of relatively large molecular structures, which are not completely digested in the oral cavity and the gastrointestinal path by digestive enzymes into monomers.

 I think, this is a very interesting work, and this manuscript is worthy of publication in your journal.

I have read this submission. I believe that I have an appropriate level of expertise to confirm that it is of an acceptable scientific standard.

# Peer review: Referee response for version 1

**DOI:** 10.5256/f1000research.15679.r35166

**Published:** 2018-07-10

**Authors:** József Prechl

**Affiliations:** 1 R&D Laboratory, Diagnosticum zrt, Budapest, Hungary

The authors measure immunoglobulin containing complexes by dynamic light scattering and the relationship between dietary intake and the presence, amount and quality of these complexes. Experiments are carried out using rats and by obtaining blood from human volunteers. The stated aim of the study is the monitoring of quantity and quality of immune complexes following food intake.

 Methodology

 Protein A binds either poorly or not at all rat immunoglobulins, so it was not a good choice for the experiment. Therefore the fact that complexes with a hydrodynamic radius of 200-300 nm are removed from rat serum is only indirect and weak evidence that these complexes are immune complexes. Even if these complexes contain immunoglobulins the specific interactions between antibody and antigen should be proved in order to be called immune complexes.

 Results

 Figure 1A and Figure 2C are identical, redundance could be reduced by merging these figures.

 Protein A treatment seems to alter composition not only by removing large complexes but also by increasing the relative contribution to light scattering of 81-109 nm sized complexes. This is not addressed by the authors. Similar complexes are present in the IVC sample (Figure 2D) of fed versus fasted rats (Fig 2B).

 How was the fraction of particles matching the ICs in human heparinized plasma determined? Figure 3 and Table 1 is based on the identification of this fraction, yet it is not defined under Methods.

 Discussion

 The first two paragraphs on page 11 provide a general overview of secreted IgA but the authors do not explain how that is related to their findings; in the present context it is not relevant and could be omitted.

 In Table 2 it is not clear whether + sign means OR or AND; IgG1+IgG3 seems to refer to complexes containing either IgG1 OR IgG3, while IgG1+IgA seems to refer to double positive (IgG1 AND IgA) complexes. Figure 4B-D-F suggests that complexes contain exclusively either IgG1 or IgA, these isotype specific monoclonals reacting with roughly half-half of the complexes. This is very unusual for immune complexes where  different isotypes against the same antigen are usually present at the same time.

 Conclusions

 The authors claim that the molecular and cellular "mechanisms of the formation and utilization of food immune complexes in humans and rodents do not match completely". The experimental and methodological approach was different in rodents and humans, therefore such a conclusion cannot be reached. Kinetics was not followed in rats that were fed ad libitum, evidence is only indirect via the use of two sampling sites (vena portae, vena cava inferior) in contrast to temporal monitoring in humans. No cellular measurements were done at all, therefore conclusion on cellular mechanisms cannot be drawn.

 Minor formal comment

 Throughout the figures cyrillic "nm" is used for nanometer, please replace with latin nm.

 The topic, how ingested food components are recognized by preformed immunoglobulins and how this recognitions affects food uptake and immunological tolerance and metabolism in general, is very timely and interesting. The article has several weak points though that need to be improved for solid and convincing results.

I have read this submission. I believe that I have an appropriate level of expertise to confirm that it is of an acceptable scientific standard, however I have significant reservations, as outlined above.

# Peer review: Referee response for version 1

**DOI:** 10.5256/f1000research.15679.r35398

**Published:** 2018-07-03

**Authors:** Boris A. Margulis

**Affiliations:** 1 Institute of Cytology of Russian Academy of Science, Saint Petersburg, Russian Federation

The study of Landa and others aims to understand how food digestion process affects circulating immune complexes, CIC. Since the amount of immune complexes increases in biological fluids as the result of various disorders, including autoimmune diseases, viral and bacterial infections and malignancies, the appearance and precipitation of the particles need more careful investigation, particularly as applied to processes of normal physiology. The idea of the study was to measure the content of CICs after food digestion, process believed to increase the particles’ number in plasma. The authors employed the method of dynamic light scattering (DLS) to analyze the size of CIC particles, and the accuracy of this method was proved in studies of ICs from rats and humans after food ingestion. To identify Ig participating in CIC assembly Sepharose microspheres covered with Protein A were used, and both methods allowed to estimate the dynamics of CIC accumulation and lowering in blood. Using this combination of techniques the authors identified the fraction of particles with the size of 200-300 nm as food CIC. The authors stated that most of CIC in a process of digestion appear in blood from intestine and then they are quickly and completely utilized by the liver. In contrary, in humans the accumulation and removal of CICs from blood occurs more slowly than in rodents and takes approximately 12 hours. The cause of such delay is explained by authors by an incomplete digestion of larger molecular structures persisting in food. To define the spectra of Igs in CIC the authors used monoclonal antibodies generated in house. The changes in Ig classes during CIC utilization lasting 7-9 hours after ingestion were strong and it was found that instead of initial predominance of IgGs IgA become prevalent at the end of that period. This change may be hypothetically associated with the necessity of maintaining of immune system in activated state after food consumption. The study of Landa *et al* evokes several questions and one of the latter concerns the  experimental approval of dependence of CIC content and composition on meal volume and/or composition. To conclude, the authors made a big work; they implemented a novel technique, DLS, for the analysis of immune complexes and obtained interesting results concerning particularly the long-living CIC in humans resulting from food consumption.

I have read this submission. I believe that I have an appropriate level of expertise to confirm that it is of an acceptable scientific standard.
